# Theory and Applications of Surface Plasmon Resonance, Resonant Mirror, Resonant Waveguide Grating, and Dual Polarization Interferometry Biosensors

**DOI:** 10.3390/s101109630

**Published:** 2010-11-01

**Authors:** Hikmat N. Daghestani, Billy W. Day

**Affiliations:** 1 Department of Structural Biology, University of Pittsburgh, BST3 10017, 3501 Fifth Ave, Pittsburgh PA, 15213, USA; E-Mail: hnd3@pitt.edu; 2 Departments of Pharmaceutical Sciences and of Chemistry, University of Pittsburgh, BST3 10017, 3501 Fifth Ave, Pittsburgh PA, 15213, USA

**Keywords:** optical biosensor, surface plasmon resonance, resonant mirror, resonance waveguide grating, dual polarization interferometry

## Abstract

Biosensors have been used extensively in the scientific community for several purposes, most notably to determine association and dissociation kinetics, protein-ligand, protein-protein, or nucleic acid hybridization interactions. A number of different types of biosensors are available in the field, each with real or perceived benefits over the others. This review discusses the basic theory and operational arrangements of four commercially available types of optical biosensors: surface plasmon resonance, resonant mirror, resonance waveguide grating, and dual polarization interferometry. The different applications these techniques offer are discussed from experiments and results reported in recently published literature. Additionally, recent advancements or modifications to the current techniques are also discussed.

## 1. Introduction

Biosensors that rely on rapid and portable screening techniques have been of interest to identify harmful toxins for food safety [1,2] or to detect chemical or biological agents that could be used in bioterrorism [3–5]. Biosensors are also of interest for research purposes in areas of biophysics and pharmaceutical sciences. The obvious advantage biosensors offer over many other biophysical techniques is that it is label-free, eliminating the need for fluorescent, chemical, or radiolabeled tags. In addition, biosensor technologies are relatively easy to use and offer real-time data collection so that different biochemical interactions can be monitored. Biosensors have several applications for illuminating explanations to questions arising from the study of macromolecular interactions [6] and the binding of small molecules to surfaces with immobilized biological molecules [7–9]. The types of biosensor arrangements vary greatly and have been previously reviewed. Examples of biosensor types include electrochemical [10,11], carbon nanotube field effect [12,13], and optical [14]. Within each of these individual types, there are many variations in the instrument designs. This review will discuss the basic theory of several of the most commonly used commercial optical biosensors in the field and provide an update on the current applications each of these techniques offer.

## 2. Surface Plasmon Resonance Biosensors

**Figure 1.**
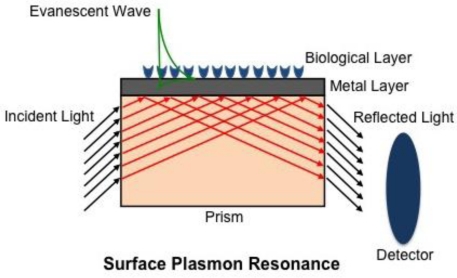
Schematic of a surface plasmon resonance biosensor (Kretchmann configuration). Light reflected from a prism induces an evanescent field in both the metal and dielectric (biological) layer, with the field being greater in the latter. Light is then reflected out of the prism and a detector records the angle at which resonance is satisfied.

**Figure 2.**
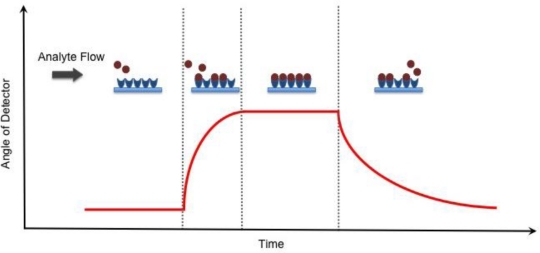
Detection of binding events for SPR and RM. As analyte begins to flow over the sensing layer and binds to substrate, the angle of reflectivity that satisfies the resonance condition will change accordingly until it reaches saturation and all the binding sites have been occupied. The dissociation of analyte from the substrate causes the angle of the detector to return back to baseline once all the analyte has been completely removed.

Surface plasmon resonance (SPR) was first demonstrated by Otto in 1968 [15], but was not made commercially available for biomolecular interaction applications until the fall of 1990 by Biacore^®^ (GE Healthcare) [16]. As a starting point, we will consider surface plasmon polaritons (SPP), which are electromagnetic (EM) modes or oscillations arising from the interaction of light with mobile surface chargers in a metal (typically gold or silver) [15]. SPPs are transverse magnetic (TM) waves that propagate along the interface between materials with negative and positive permittivities (e.g., a metal/dielectric layer). According to the Drude model, the dispersion relation *β* of an SPP, which essentially correlates the relationship between the wavevector along the interface and the angular frequency *ω*, can be described by

(1) $$\beta =\frac{\omega }{c}\sqrt{\frac{{ɛ}_{m}{ɛ}_{d}}{{ɛ}_{m}+{ɛ}_{d}}}$$

where *c* is the speed of light in a vacuum, while *ɛ_m_* and *ɛ_d_* are the permittivity of a metal and a dielectric material, respectively. The real part of Equation (1) determines the SPP wavelength, while the imaginary part determines the propagation length of the SPP along the interface, which is responsible for the evanescent field [17]. Although the EM field of an SPP decays evanescently into both the metal and dielectric medium, the majority of the field is present in the dielectric medium due to increased damping in the metal [17], Figure (1). As a result, the real part of the dispersion function is very sensitive and changes proportionally to changes in the refractive index [18]. The principle of SPR, however, only occurs when the light’s wavevector component parallel to the metal surface matches that of the SPP. This condition is only satisfied at distinct angles of incidence, appearing as a drop in the reflectivity of incident light [17,18]. SPR biosensing relies on the principle that any changes on the dielectric sensing surface will cause a shift in the angle of reflectivity, followed by a detector, in order to satisfy the resonance condition as depicted in Figure (2).

After Otto demonstrated the ability to excite SPPs with his proposed configuration, a number of other configurations followed suit including prism coupling (Kretschmann configuration; also referred to as attenuated total reflection (ATR)) [18,19], waveguide coupling [20], grating coupling [21], and fiber optic coupling [22]. In the case of the most commonly used Kretschmann configuration, incident light passes through a prism with a high index of refraction causing the light to internally reflect at the metal/prism boundary. The total internal reflection creates an evanescent wave that penetrates the thin metal layer and propagates along the metal/prism interface. The angle of incident light is varied in order to match the evanescent wave propagation rate with the propagation rate of the SPP [19]. Grating coupling may also be used to excite SPPs by stimulating a periodic metal diffraction layer with incident light so that the propagation constant also matches that of the metal/dielectric surface [18,21]. Waveguide coupling relies on exciting SPPs when the guided light and the SPPs are phase matched [23]. Regardless of the configuration, environmental changes in the dielectric medium cause an alteration to the phase, amplitude, polarization or spectral distribution of the incident light, which can be attributed to changes in the propagation constant and, hence, changes in the refractive index are detected in real time. Piliarik and Homola [24] recently presented a theoretical analysis evaluating the sensitivity of SPR detection, suggesting that many of the current systems, regardless of their instrumental arrangement, very nearly approach their theoretical limits.

The most common use for SPR sensing is to evaluate protein-ligand [25], protein-protein [26], or nucleotide hybridization [27] events. Since it is typically not advantageous to directly deposit biological molecules onto surfaces, especially surfaces of inert metals such as silver or gold, surface functionalization can be used to create a more functionally active environment and reduce non-specific binding on the surface. Advancements in surface chemistry have allowed researchers to easily customize a sensing surface to their particular needs. One of the most commonly used surfaces includes those prepared through amine chemistry, such as *N*-hydroxysuccinimide (NHS)-derivatized surfaces [28–30] that nonspecifically bind to the nucleophilic amino groups of peptides and proteins. Similarly, maleimide and other thiol-reactive groups [30,31] are useful for binding proteins containing surface reactive cysteines. Pegylation (polyethyleneglycol) [30,32] is another surface functionalization method that is often used in biosensor applications. Additionally, surfaces have also been mimicked to resemble lipid bilayers for studies involving membrane proteins [33,34]. Protein-carbohydrate interactions can also be monitored by glycan-modified surfaces [35,36]. By taking advantage of the extremely strong affinity of biotin for avidin or streptavidin, biotinylated surfaces [37] can be particularly useful for capturing labeled proteins, as we have recently demonstrated [38]. Nickel nitrilotriacetic acid (Ni-NTA)-derivatized surfaces [39,40] are also convenient for specific capturing of proteins that have been genetically engineered with an N- or C-terminal polyhistidine tag, a common affinity moiety used during protein expression and purification processes.

High contrast SPR microscopy or imaging was first described by Rothenhausler and Knoll [41] and was seen as a method to increase the throughput of standard SPR biosensors [42–44], but suffered from reduced sensitivity compared to conventional SPR. Advances in microfabrication and micromachining techniques have assisted in the development of lab-on-chip sensors with better sensitivity and greater numbers of sample chambers within a single chip. These advancements have played a role in SPR imaging developments for high throughput biosensor screening. Piliarik *et al.* [45] developed a more sensitive SPR imaging sensor that combines polarization contrast and special SPR multilayer structures capable of screening 108 samples simultaneously at a concentration as low as 500 ng/mL and with minimal crosstalk between chambers. A chip proposed by Ouellet *et al.* [46] demonstrated the ability to simultaneously monitor multiple ligands against different analytes and at different concentrations by using a parallel 264-microarray chamber with the aid of a high resolution CCD camera. In addition to the increased number of events detected, the microfluidics was designed for small reaction volumes (as low as 700 pL), reducing unnecessary sample consumption. The authors also demonstrated the ability to recover samples after SPR measurements with minimal cross-contamination.

In recent years, the information obtained from SPR has also been used to complement the information obtained from mass spectrometry (MS), providing both quantitative and qualitative information [47]. The combined use of SPR and MS can be used for functional proteomic screening, identifying protein-protein interactions and further characterizing domains involved in the interactions [48,49]. This technique can also be used for screening of a number of toxins for their ability to bind a particular ligand, followed by MS analysis to determine the chemical composition of the small molecules [50,51]. Another application involves searching for and characterizing enzyme inhibitors [52]. Some investigators have attempted to elute samples off of and collect samples directly from the sensor and then analyze the eluates by matrix-assisted laser desorption ionization (MALDI) time-of-flight (TOF)-MS [53,54]. Without taking strenuous care, such sample transfer techniques can lead to a great amount of sample loss between steps and can be very time consuming unless more efficient techniques, such as the chip proposed by Ouellet and colleagues [46], can be put into such practice. Other ways to minimize sample losses include applying MALDI matrix directly onto the sample sensor, which is then physically secured onto a MALDI target to analyze samples directly without an elution step. This technique, however, is destructive to the sample chip and introduces sources of error since not all chips are identical in terms of thickness (Δthickness = Δdistance from the MALDI-TOF-MS ion detector, and therefore ΔTOF). Natsume *et al.* [55] showed it possible to collect the samples used in a Biacore SPR instrument by trapping the sample into a reverse-phase (RP) capillary column placed in tandem after the sample sensor. In this configuration, once the desired measurements were obtained from the SPR, the sample flow was started so that buffer eluted the sample from the sensor chamber into the RP capillary column. After the sample was collected on the RP capillary column, the column was transferred to a liquid chromatography system to separate sample constituents followed by ESI-MS analysis [55]. It is not feasible to flow samples from an SPR sensor directly to a MS because salts and stabilizers that are present in buffers can often wreak havoc on an ESI-MS system by damaging the ESI needle and decrease the quality of the MS spectra (by, e.g., diluting signals across several adduct species, ion suppression, and increasing the noise-to-signal ratio) [56]. An alternative method is to use an ultra-rapid desalting technique consisting of a microchannel laminar flow device connected online with an ESI-MS [57]. From these examples, it is evident that combining biosensors with MS offers a promising future at providing immediate structural and behavioral information about potential biologically important agents (therapeutics, toxins, *etc.*) in a relatively short period with minimal sample loss.

## 3. Resonant Mirror Biosensor

**Figure 3.**
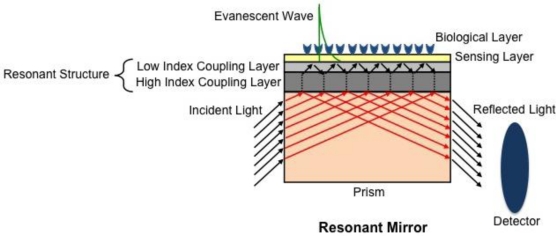
Schematic of a resonant mirror biosensor. Light reflected from a prism is coupled to a resonant structure (low and high index coupling layers) to produce an evanescent wave at the sensing surface. Light is then reflected out of the prism and a detector records the angle at which resonance is satisfied (adapted from [60] with permission from publisher).

The resonant mirror (RM) setup is a leaky waveguide structure that first became commercially available as IAsys in 1993 by Fisons Applied Sensor Technologies [16]. Although the commercial availability of this instrument was recently discontinued, it is still important to note its application and contribution to the field. The RM configuration is similar to SPR’s Kretschmann configuration, but differs in that RM relies on coupling of incident light through a prism with a high-index dielectric layer, rather than a metal surface, Figure (3). This replacement combines the simple structure of SPR systems with the enhanced sensitivity of waveguide structures to produce sharper resonance peaks than SPR [58], thereby increasing the sensitivity of the technique. As light passes through the prism to a low-index medium, it couples with the high-index resonant layer, thereby allowing total internal reflection to occur at the boundary of the sensing layer. Similar to SPR, resonance only occurs when the angle of the incident light and the resonant modes in the high-index layer are phase-matched, resulting in strong reflection at the output. Any change in the refractive index of the biological layer at the surface corresponds to a change in the angle of light that satisfies this resonance condition [59,60]. Although the waveguide structure of the RM allows for both TM and transverse electric (TE) resonances (with different angles) to occur, generally only one is physically measured since TM and TE modes diverge when adjusting the thickness of the resonant structure for optimal sensitivity [60].

Identical to SPR, RM has been used to monitor many different molecular interactions of macromolecules [61–64] and has parallel capabilities in terms of surface modifications. The cuvette structure of the RM biosensor, however, provides an advantage over flow through microfluidic systems commonly used in SPR when sample conservation is imperative. Use of a stirring bar in the cuvette is also helpful since the constant mixing limits mass transport effects [16].

## 4. Resonant Waveguide Grating Biosensor

**Figure 4.**
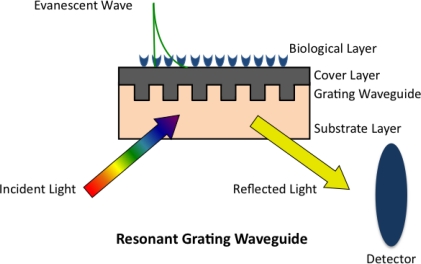
Schematic of a resonant grating waveguide biosensor. Broadband light is incident from either the substrate or cover layer side of the structure, which then diffracts and couples into the grating waveguide structure. A detector records the wavelength of the narrowband light reflected at which resonance is satisfied (adapted from [68] with permission from publisher).

Although diffraction grating was a phenomenon described over a century ago [65], its application in sensing was not employed until the early 1980’s when Tiefenthaler and Lukosz applied grating couplers for gas [66] and chemical [67] sensing. In 2002, Cunningham *et al.* [68] demonstrated the use of a resonant diffractive grating surface to monitor biochemical binding events, which was commercialized as BIND^®^ by SRU Biosystems. The resonant waveguide grating (RWG) biosensor is also based on a leaky mode waveguide structure. A subwavelength structured surface is introduced by sandwiching a two-dimensional grating between a substrate and a cover layer that fills the gaps between the gratings, which in turn creates a waveguide when the effective index of refraction of the grating is greater than the substrate or the cover [68]. Incident light, from either side of the grating [67], propagates through and couples into the waveguide by means of the grating, resulting in a narrowband of reflected or transmitted wavelengths detected as the output [69], Figure (4). Similar to SPR and RM, any change in the biological or sensing layer will cause a change in the reflected or transmitted wavelength [69,70]. Corning Inc., has also introduced its Epic^®^ version of the RWG biosensor and both companies have made modifications to their original designs to increase sensitivity and by offering 96-, 384-, and 1536-well plates suitable for high throughput screening [69,71]. Others have performed theoretical analyses on RWG structures to optimize the design and fabrication of grating structures in an attempt to improve sensitivity [72]. RWG biosensors are capable of monitoring the binding of small molecules to proteins [68,73,74] as with SPR and RM, but have most notably been used to monitor mass redistribution of proteins and organelles of live cells upon treatment with test agents [70,75–78]. Changes in cell adhesion and extracellular matrix components play an important role in cell development and migration and it is evident that certain changes in cell adhesion also contribute to a number of diseases [79]. The ability of RWG biosensors to monitor changes of cell adhesion of live cells in real time make it an attractive tool in drug discovery. A drawback of evaluating cells with biosensors arises due to the large size of cells (several microns) and the limited penetration depth of an evanescent wave (∼100 nm), results can be misleading since observations are only made to a limited portion of the cell [75].

## 5. Dual Polarization Interferometry Biosensor

**Figure 5.**
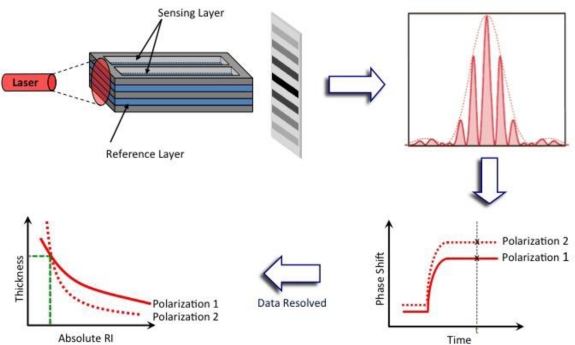
Schematic of a DPI sensor chip and the interference pattern produced when light is applied onto the side of a chip. The phase shift of the fringes (TM and TE) are recorded in real time and data is resolved, where only one value of thickness and absolute refractive index at any given time-point *t* will satisfy Maxwell’s equations of electromagnetism for both TM and TE polarizations (adapted from [81] with permission from publisher).

Dual polarization interferometry (DPI) is another evanescent technique that has seen a large increase in interest by the scientific community over the past decade since the technique was first commercialized in 2000 by Farfield Group, Ltd. DPI utilizes a waveguide structure that consists of a stack of dielectric layers with reference and sensing layers separated by a layer of cladding that mimics Young’s 2-slit experiment in optics [80]. A top dielectric layer is etched to reveal the sensing layer so that two separate channels can be present on a single sensor chip, Figure (5). Light from a laser is passed through the sandwiched waveguide structure and an interference pattern is detected on the opposing side by a CCD camera. Any changes in refractive index that take place on the sensing layer alter the phase position of the fringes relative to the reference layer and are detected in real time,

(2) $$\Delta \phi =kL^{\prime }\Delta n_{s}$$

where Δϕ is the change in the phase position of a fringe, *k* is the propagation constant, *L′* is the pathlength and is constant, and *Δn_s_* is the effective change in the refractive index of the sensing waveguide [80].

Unlike SPR, which utilizes only the TM mode, DPI takes advantage of measuring both the TM and TE polarizations [80–82]. Maxwell’s equations of electromagnetism for a system of uniform multiple dielectric layers are employed to provide the absolute effective index for both the TM and TE waveguide modes determined from the refractive index and thickness of each layer from each polarization [80]. This ultimately gives the relationship between changes in the effective index of refraction *Δn_eff_* of the waveguide in each mode and changes of thickness of the adsorbed layer *t_ad_* (in nm)

(3) $$\Delta n_{\mathrm{eff}}=\left(\frac{\partial n_{\mathrm{eff}}}{\partial t_{\mathrm{ad}}}\right)\Delta t_{\mathrm{ad}}+\left(\frac{\partial n_{\mathrm{eff}}}{\partial n_{c}}\right)\Delta n_{c}$$

where *Δn_c_* is the change in refractive index of the medium covering the waveguide (*i.e.*, buffer) [67,83]. Changes to the adsorbed layer will result in a change to the effective index of each mode that can satisfy a continuous distribution of thickness and refractive index values with only one unique solution that satisfies both the TM and TE modes. In addition, the molar surface coverage Γ (in nm^2^·molecule^−1^) can be related to the thickness of the adsorbed layer

(4) $$\Gamma =\frac{n_{\mathrm{ad}}-n_{c}}{{\mathrm{dn}}_{\mathrm{ad}}/\mathrm{dC}}t_{\mathrm{ad}}$$

where *n_ad_* is the refractive index of the adsorbed layer and *C* is the concentration. Consequently, the density *ρ* (in g·cm^−3^) of sample on the surface can be calculated for biological samples with known molecular weight *M* [80] since molar surface coverage can also be written as

(5) $$\Gamma =\frac{\rho }{M}t_{\mathrm{ad}}$$

The use of both polarizations to determine effective refractive index and thickness values is clearly a great advantage over SPR, RM, RWG, and other optical biosensor techniques that only report relative changes of refractive index obtained from only one polarization. Swann *et al.* [81] proposed an elegant matrix that assists interpreting DPI data by correlating the different parameters (thickness, density, mass coverage) calculated from both the TM and TE responses. This type of detailed information can be extremely helpful for characterizing the conformational changes of macromolecular interactions [81,84,85] and the design of surfaces for optical biosensors [38,86].

Before any of the above calculations are performed, each individual chip must be calibrated. Sample injections of degassed 4:1 (w/w) water-ethanol followed by deionized water are typically used to calibrate individual chips because of their known index of refractions [80]. One disadvantage of DPI is that an experiment must be performed continuously in order to follow the phase shift of the projected interference pattern so that thickness and refractive index values can be computed. This can hinder calibration results if a chip requires *ex situ* modification throughout an experiment. A solution to this issue, however, was recently proposed by modifying the channel so that multiple pathlengths are measured [87]. With this adjustment, the number of 2π cycles of the phase shift can be determined if the chip is removed from the instrument, thereby allowing *ex situ* modification of the chip without the loss of any information.

In addition to the same applications of SPR and RM [88,89], DPI has proven to be a powerful technique for characterizing structural dimensions of proteins [90] and has recently been shown to be an instrumental tool for characterization of membrane/liposome structure and mimetics [91–94]. Another unique application to DPI that recently emerged is the ability to monitor early stages of protein crystallization processes by measuring light loss from the waveguide caused by changes in lateral surface structure [95]. Not only does this provide insights into the mechanism of protein crystallization, but also has the potential to assist crystallographers in the optimization of conditions and times required for successful protein crystallization. More recently, information from phase measurements has been supplemented by optical extinction measurements due to light absorption to provide additional information of DNA-small molecule interactions [96].

It should be noted that any of the above mentioned biosensors can be used to determine the kinetics of biomolecular interactions from the rates of association *k_ass_* and dissociation *k_diss_* of a substrate-ligand complex by monitoring the change in response of binding as a function of time and concentration

(6) $$\frac{d\left[\mathrm{SL}\right]}{dt}=k_{\mathrm{ass}}\left[S\right]\left[L\right]-k_{\mathrm{diss}}\left[\mathrm{SL}\right]$$

where [S] and [L] are the concentrations of free substrate and ligand, respectively, while [SL] is the concentration of the formed complex [97]. At equilibrium, the association of ligand to the surface can be followed by the pseudo first order equation

(7) $$R_{t}=\frac{R_{\text{max}}k_{\mathrm{ass}}\left[L\right]}{k_{\mathrm{ass}}\left[L\right]+k_{\mathrm{diss}}}\left(1-e^{-\left(k_{\mathrm{ass}}\left[L\right]+k_{\mathrm{diss}}\right)t}\right)$$

where *R_t_* is the response of the detector at a given time and *R_max_* is the maximal response signal upon saturation [98]. Typically, experiments are performed by varying the amount of ligand added to the substrate, producing curves with different observed rate constants *k_on_*. *By plotting the k_on_* against the varying concentration of ligand, Equation (8), a straight line is typically produced with a slope of *k_ass_* and y-axis intercept of *k_diss_* [99].

(8) $$k_{\mathrm{on}}=k_{\mathrm{ass}}\left[L\right]+k_{\mathrm{diss}}$$

This information can then lead to the association *K_a_* and dissociation *K_d_* equilibrium constants because of the below relationship [97]

(9) $$K_{a}=\frac{1}{K_{d}}=\frac{k_{\mathrm{ass}}}{k_{\mathrm{diss}}}$$

With a similar type of analysis as described above, kinetic values from second order reactions may also be calculated as previously described [98,99].

## 6. Conclusions and Future

Biosensors offer label free detection of biomolecular interactions with applications in environmental safety, bioterrorism, biomedical research and drug discovery. Several designs are capable of detecting biomolecular interactions. Surface plasmon resonance, resonant mirror, resonant waveguide grating, and dual polarization interferometry biosensors are commonly used techniques with commercial availability. SPR, being the most widely used technique in the field, has provided researchers with a wealth of information ranging from evaluation of many different biomolecular interactions to advances in sensor design. Progress in chip design has allowed for smaller sample volumes, not only to save valuable samples, but also to increase rates of the reactions by reducing diffusion distances. Advances in computer automation and the software to analyze the exquisite data that arise from the discussed methods has also played an important role in greater reproducibility and easier sample handling with all four of the biosensor types capable of employing autosamplers. Although SPR is one of the most commonly used biosensor techniques, the cuvette structure of RM offers an advantage over both SPR and DPI because of its ease of use and ability to reduce sample consumption compared to microfluidic devices. Since the IAsys system has recently been discontinued, RWG structures offered by SRU Biosystems and Corning, Inc. offer an alternative method that takes advantage of the increased sensitivity of a waveguide structure in addition to the high throughput applicability from the multi-well plates available. On the other hand, DPI offers a unique avenue of monitoring biomolecular interactions and the detailed structural changes that take place during these interactions. Overall, biosensors are immensely useful in many different applications and future research aims at improving the sensitivity and throughput of these devices for greater reproducibility and applicability to larger sets of data acquisition.
